# Vulvar lichen sclerosus in pregnancy: Unaddressed needs and systematic review

**DOI:** 10.1002/ski2.281

**Published:** 2023-09-04

**Authors:** Britt Debaene, Sander Vandeweege, Hans Verstraelen

**Affiliations:** ^1^ Faculty of Medicine and Health Sciences Ghent University Ghent Belgium; ^2^ Department of Obstetrics & Gynaecology Ghent University Hospital Ghent Belgium; ^3^ Department of Human Structure and Repair Faculty of Medicine and Health Sciences Ghent University Ghent Belgium

## Abstract

Women with vulvar lichen sclerosus (vLS) often express their concern over disease course, treatment, and complications relating to vLS in pregnancy and following childbirth. We used such patient concerns as study objectives in a systematic literature research. Although few, mostly low‐quality studies have been published on that matter, and albeit the vast majority of women will have stable vLS during pregnancy, available data also suggest that in a small but significant proportion of patients, pregnancy and delivery may complicate the course of vLS and vice versa.

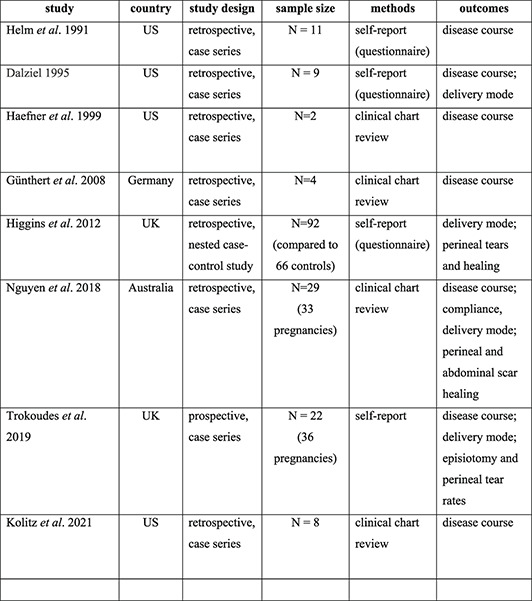

Dear Editor, vulvar lichen sclerosus (vLS) is a chronic inflammatory skin condition that may affect the anogenital area in women of any age. Treatment of vLS with high‐potency topical corticosteroids (TCS) and emollients/moisturizers should be assumed for life, regardless of symptoms, and further adjusted to disease activity.^1^ Apart from providing symptomatic relief, long‐term maintenance therapy is key in maintaining disease remission, in reducing the risk of scarring and loss of vulvar anatomy, and, notably, in minimising the risk of squamous cell carcinoma in vLS.^2^, ^3^

In clinical practice we perceive a defined patients' needs however, for information on disease course in relation to pregnancy and childbirth. Obstetric health care providers in turn may not be familiar with vLS. We therefore listed a set of topical patients' questions and concerns, and further sought to provide answers through a systematic literature search in line with the PRISMA statement,^4^ and according to a preregistered protocol (Prospero CRD42023411358).^5^ Specifically, we searched MEDLINE, EMBASE, and Web of Science, without limits or language restriction, up to 12 February 2023 and further cross‐checked reference lists of selected studies. Studies were considered eligible if these addressed at least one of the following questions: Does pregnancy alter the disease course of vLS?; Does or may vLS necessitate delivery by primary caesarean section?; Is there an increased risk for perineal tears with vLS?; May vLS delay or complicate healing of episiotomy and/or perineal tears?; and, does vLS worsen following delivery? Eligibility screening of records retrieved was performed independently by all authors. Risk of bias was assessed through use of the appropriate JBI tool for the respective study designs.^6^, ^7^

In this manner, we retrieved eight studies, published between 1991 and 2021, with data on pregnancy and/or delivery in a total of 177 vLS patients (sample size range, 2–92) (Table 1). Except for a single nested case‐control study, all studies were uncontrolled and except for one prospective study, all studies had a retrospective case series design.

**TABLE 1:** Overview of studies on vulvar lichen sclerosus in women giving birth.

Study	Country	Study design	Sample size	Methods	Outcomes
Helm et al. 1991^10^	USA	Retrospective, case series	*N* = 11	Self‐report (questionnaire)	Disease course
Dalziel 1995^11^	USA	Retrospective, case series	*N* = 9	Self‐report (questionnaire)	Disease course; delivery mode
Haefner et al. 1999^12^	USA	Retrospective, case series	*N* = 2	Clinical chart review	Disease course
Günthert et al. 2008^13^	Germany	Retrospective, case series	*N* = 4	Clinical chart review	Disease course
Higgins et al. 2012^14^	UK	Retrospective, nested case‐control study	*N* = 92 (compared to 66 controls)	Self‐report (questionnaire)	Delivery mode; perineal tears and healing
Nguyen et al. 2018^15^	Australia	Retrospective, case series	*N* = 29 (33 pregnancies)	Clinical chart review	Disease course; compliance, delivery mode; perineal and abdominal scar healing
Trokoudes et al. 2019^16^	UK	Prospective, case series	*N* = 22 (36 pregnancies)	Self‐report	Disease course; delivery mode; episiotomy and perineal tear rates
Kolitz et al. 2021^17^	USA	Retrospective, case series	*N* = 8	Clinical chart review	Disease course

Both worsening of vLS, not otherwise specified (reported in two studies, with worsening in 5 of 11, and 2 of 8 patients, respectively), as well as improvement during pregnancy (reported in three studies, with improvement in 5 of 11, 3 of 9, and 4 of 4 patients, respectively) was reported, while absence of an observed effect of pregnancy on vLS was by far the most common outcome across studies (reported in four studies, with an absence of effect on vLS in all 33 pregnancies in 29 patients, in all 36 pregnancies in 22 patients, and in 5 of 9, and 6 of 8 patients in two previously listed studies, respectively). Overall, disease course of vLS during pregnancy was reported in 7 out of 8 studies, relating to a total of 85 women, of which 12 patients experienced improvement, 65 women had stable vLS, and 7 patients reported worsening. Notably, in 5 patients, vLS reportedly developed, either was first diagnosed in pregnancy (reported in two studies, with onset and/or first diagnosis in 1 of 11, and 4 of 29 patients, respectively). Delivery through caesarean was attributed to vLS (in one patient owing to non‐compliance) in a single patient in two studies each (1 of 11, and 1 of 29 patients, respectively). In only one patient was there a suggestion of vLS‐related risk for perineal tears (1 of 9 patients, relating to 4 deliveries). One study also mentioned delayed healing of a third degree tear in one patient not under treatment (1 of 22 patients), while in another study development of vLS/koebnerization in the area of the perineal scar occurred (1 of 29 patients). Finally, worsening of vLS in the postpartum was mentioned only once with regard to three pregnancies in one woman (one study, 1 of 9 patients).

Overall, there were few studies on vLS in pregnancy. Studies were rather small, mostly retrospective and uncontrolled, rarely used standardized measures such as TCS need, and often provided little detail on treatment altogether, and therefore particularly prone to bias. Hence, no meaningful estimates can be made on the incidence of outcomes addressed. Nonetheless, the small literature base retrieved, suggests that in an apparently small proportion of patients, pregnancy and delivery may complicate the course of vLS and vice versa. Specifically, vLS was reported to possibly worsen in pregnancy, and in a few cases, to necessitate caesarean delivery, to underlie perineal trauma, and to delay or complicate perineal wound healing. While it cannot be inferred from these data whether this is also preventable, a number of complications of vLS are in fact largely prevented by long‐term topical treatment.^2^, ^8^ Furthermore, cases were described with onset either first diagnosis in pregnancy suggesting that prenatal care may provide a window of opportunity to early diagnosis and treatment of vLS.

In conclusion, while there is a defined need for prospective data on vLS in pregnancy, currently available data do suggest that in a majority of vLS patients under treatment, pregnancy does not complicate vLS and vice versa. Nonetheless, early diagnosis and proper treatment remain key, also in pregnancy, in order to possibly avoid negative outcomes relating to vLS in pregnancy. Recently published guidelines on that matter,^9^ as well as efforts to increase vulvar awareness among obstetric health care providers may further benefit women with vLS during and following pregnancy.

## CONFLICT OF INTEREST STATEMENT

None to declare.

## AUTHOR CONTRIBUTIONS

**Britt Debaene**: Data curation (supporting); formal analysis (lead); methodology (supporting); project administration (lead); validation (supporting); visualization (lead); writing—review & editing (equal). **Sander Vandeweege**: Data curation (supporting); formal analysis (lead); methodology (supporting); project administration (lead); validation (supporting); visualization (lead); writing—review & editing (equal). **Hans Verstraelen**: Conceptualization (lead); data curation (lead); methodology (lead); supervision (lead); writing—original draft (lead); writing—review & editing (lead).

## ETHICS STATEMENT

Not applicable.

## Supporting information

Supporting Information S1

Supporting Information S2

## Data Availability

The data that supports the findings of this study are available in the supplementary material of this article.
