# Supplementary material for: Vulvar lichen sclerosus in pregnancy: Unaddressed needs and systematic review
Source: Skin Health Dis. 2023 Sep 4;3(5):e281. doi: 10.1002/ski2.281 (PMC10549801; doi:10.1002/ski2.281)
Supplement: Supplementary file 1 — Supporting Information S1 [file SKI2-3-e281-s001.pdf]

PRISMA 2020 flow diagram for new systematic reviews which included searches of databases, registers and other sources

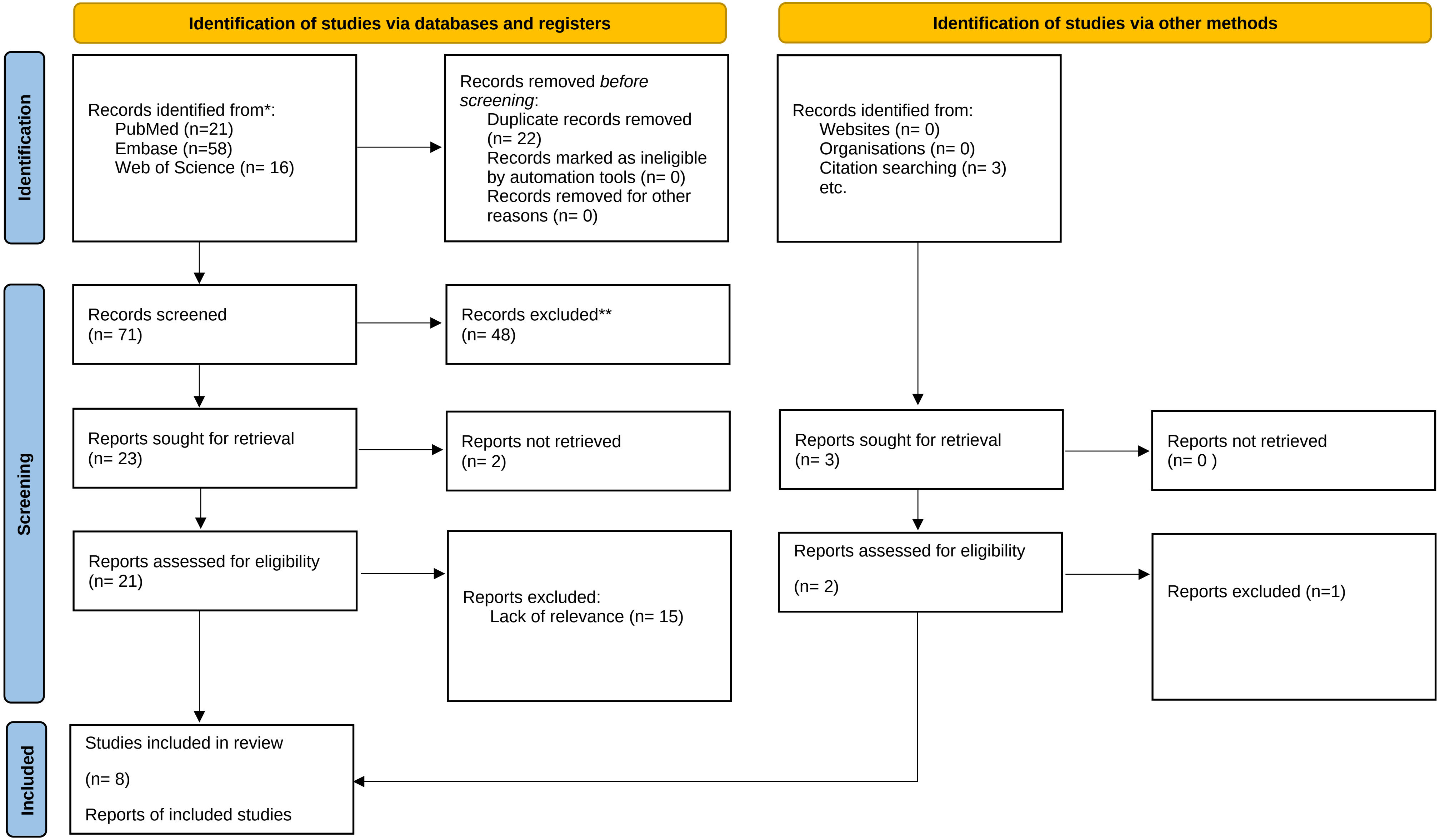

*Consider, if feasible to do so, reporting the number of records identified from each database or register searched (rather than the total number across all databases/registers).
**If automation tools were used, indicate how many records were excluded by a human and how many were excluded by automation tools.

From: Page MJ, McKenzie JE, Bossuyt PM, Boutron I, Hoffmann TC, Mulrow CD, et al. The PRISMA 2020 statement: an updated guideline for reporting systematic reviews. BMJ 2021;372:n71. doi: 10.1136/bmj.n71. For more information, visit: http://www.prisma-statement.org/
