# Supplementary material for: Vulvar lichen sclerosus in pregnancy: Unaddressed needs and systematic review
Source: Skin Health Dis. 2023 Sep 4;3(5):e281. doi: 10.1002/ski2.281 (PMC10549801; doi:10.1002/ski2.281)
Supplement: Supplementary file 2 — Supporting Information S2 [file SKI2-3-e281-s002.pdf]

JBI Critical Appraisal Checklist for Case Series

Reviewer: Britt Debaene, Sander Vandeweege and Hans Verstraelen Date: 16/04/2023

Author: Elysha Kolitz Year: 2021 Record Number_________

	Yes	No	Unclear	Not applicable
• Were there clear criteria for inclusion in the case series?	X	☐	☐	☐
• Was the condition measured in a standard, reliable way for all participants included in the case series?	☐	☐	X	☐
• Were valid methods used for identification of the condition for all participants included in the case series?	☐	☐	X	☐
• Did the case series have consecutive inclusion of participants?	☐	☐	X	☐
• Did the case series have complete inclusion of participants?	☐	☐	X	☐
• Was there clear reporting of the demographics of the participants in the study?	X	☐	☐	☐
• Was there clear reporting of clinical information of the participants?	X	☐	☐	☐
• Were the outcomes or follow up results of cases clearly reported?	X	☐	☐	☐
• Was there clear reporting of the presenting site(s)/clinic(s) demographic information?	X	☐	☐	☐
• Was statistical analysis appropriate?	☐	☐	☐	X

Overall appraisal: Include X Exclude ☐ Seek further info ☐

Comments (Including reason for exclusion)

____

____

THE JOANNA BRIGGS INSTITUTE

JBI Critical Appraisal Checklist for Case Series

Reviewer: Britt Debaene, Sander Vandeweege and Hans Verstraelen Date 16/04/2023

Author: Klaus F. Helm Year 1991 Record Number_______

	Yes	No	Unclear	Not applicable
1. Were there clear criteria for inclusion in the case series?	X	☐	☐	☐
2. Was the condition measured in a standard, reliable way for all participants included in the case series?	☐	☐	X	☐
3. Were valid methods used for identification of the condition for all participants included in the case series?	X	☐	☐	☐
4. Did the case series have consecutive inclusion of participants?	☐	X	☐	☐
5. Did the case series have complete inclusion of participants?	X	☐	☐	☐
6. Was there clear reporting of the demographics of the participants in the study?	X	☐	☐	☐
7. Was there clear reporting of clinical information of the participants?	X	☐	☐	☐
8. Were the outcomes or follow up results of cases clearly reported?	X	☐	☐	☐
9. Was there clear reporting of the presenting site(s)/clinic(s) demographic information?	☐	X	☐	☐
10. Was statistical analysis appropriate?	☐	☐	☐	X

Overall appraisal: Include X Exclude ☐ Seek further info ☐

Comments (Including reason for exclusion)

____

____

____

© JBI, 2020. All rights reserved. JBI grants use of these tools for research purposes only. All other enquiries should be sent to jbisynthesis@adelaide.edu.au.

JBI Critical Appraisal Checklist for Case Series

Reviewer: Britt Debaene, Sander Vandeweege and Hans Verstraelen Date 16/04/2023

Author: Yvonne Nguyen Year 2018 Record Number_______

	Yes	No	Unclear	Not applicable
1. Were there clear criteria for inclusion in the case series?	X	☐	☐	☐
2. Was the condition measured in a standard, reliable way for all participants included in the case series?	☐	☐	X	☐
3. Were valid methods used for identification of the condition for all participants included in the case series?	X	☐	☐	☐
4. Did the case series have consecutive inclusion of participants?	X	☐	☐	☐
5. Did the case series have complete inclusion of participants?	X	☐	☐	☐
6. Was there clear reporting of the demographics of the participants in the study?	☐	X	☐	☐
7. Was there clear reporting of clinical information of the participants?	X	☐	☐	☐
8. Were the outcomes or follow up results of cases clearly reported?	X	☐	☐	☐
9. Was there clear reporting of the presenting site(s)/clinic(s) demographic information?	☐	X	☐	☐
10. Was statistical analysis appropriate?	☐	☐	☐	X

Overall appraisal: Include X Exclude ☐ Seek further info ☐

Comments (Including reason for exclusion)

____

____

____

© JBI, 2020. All rights reserved. JBI grants use of these tools for research purposes only. All other enquiries should be sent to jbisynthesis@adelaide.edu.au.

JBI Critical Appraisal Checklist for Case Series

Reviewer: Britt Debaene, Sander Vandeweege and Hans Verstraelen Date 16/04/2023

Author: HopeK. Haefner Year 1999 Record Number_______

	Yes	No	Unclear	Not applicable
1. Were there clear criteria for inclusion in the case series?	☐	X	☐	☐
2. Was the condition measured in a standard, reliable way for all participants included in the case series?	☐	X	☐	☐
3. Were valid methods used for identification of the condition for all participants included in the case series?	X	☐	☐	☐
4. Did the case series have consecutive inclusion of participants?	☐	☐	X	☐
5. Did the case series have complete inclusion of participants?	☐	☐	X	☐
6. Was there clear reporting of the demographics of the participants in the study?	☐	X	☐	☐
7. Was there clear reporting of clinical information of the participants?	X	☐	☐	☐
8. Were the outcomes or follow up results of cases clearly reported?	X	☐	☐	☐
9. Was there clear reporting of the presenting site(s)/clinic(s) demographic information?	☐	X	☐	☐
10. Was statistical analysis appropriate?	☐	☐	☐	X

Overall appraisal: Include X Exclude ☐ Seek further info ☐

Comments (Including reason for exclusion)

____

____

____

© JBI, 2020. All rights reserved. JBI grants use of these tools for research purposes only. All other enquiries should be sent to jbisynthesis@adelaide.edu.au.

JBI Critical Appraisal Checklist for Case Series

Reviewer: Britt Debaene, Sander Vandeweege and Hans Verstraelen   Date: 16/04/2023

Author: Katharine L. Dalziel   Year: 1995   Record Number_________

	Yes	No	Unclear	Not applicable
• Were there clear criteria for inclusion in the case series?	☐	X	☐	☐
• Was the condition measured in a standard, reliable way for all participants included in the case series?	☐	☐	X	☐
• Were valid methods used for identification of the condition for all participants included in the case series?	X	☐	☐	☐
• Did the case series have consecutive inclusion of participants?	☐	☐	X	☐
• Did the case series have complete inclusion of participants?	☐	☐	X	☐
• Was there clear reporting of the demographics of the participants in the study?	X	☐	☐	☐
• Was there clear reporting of clinical information of the participants?	☐	X	☐	☐
• Were the outcomes or follow up results of cases clearly reported?	X	☐	☐	☐
• Was there clear reporting of the presenting site(s)/clinic(s) demographic information?	☐	X	☐	☐
• Was statistical analysis appropriate?	☐	☐	☐	X

Overall appraisal: Include X Exclude ☐ Seek further info ☐

Comments (Including reason for exclusion)

© JBI, 2020. All rights reserved. JBI grants use of these tools for research purposes only. All other enquiries should be sent to jbisynthesis@adelaide.edu.au.

JBI Critical Appraisal Checklist for Case Series

Reviewer: Britt Debaene, Sander Vandeweege and Hans Verstraelen   Date: 16/04/2023

Author: Andreas R. Günthert   Year: 2007   Record Number_________

	Yes	No	Unclear	Not applicable
• Were there clear criteria for inclusion in the case series?	X	☐	☐	☐
• Was the condition measured in a standard, reliable way for all participants included in the case series?	☐	☐	X	☐
• Were valid methods used for identification of the condition for all participants included in the case series?	☐	☐	X	☐
• Did the case series have consecutive inclusion of participants?	☐	☐	X	☐
• Did the case series have complete inclusion of participants?	☐	☐	X	☐
• Was there clear reporting of the demographics of the participants in the study?	X	☐	☐	☐
• Was there clear reporting of clinical information of the participants?	X	☐	☐	☐
• Were the outcomes or follow up results of cases clearly reported?	X	☐	☐	☐
• Was there clear reporting of the presenting site(s)/clinic(s) demographic information?	☐	X	☐	☐
• Was statistical analysis appropriate?	☐	☐	X	☐

Overall appraisal: Include X Exclude ☐ Seek further info ☐

Comments (Including reason for exclusion)

© JBI, 2020. All rights reserved. JBI grants use of these tools for research purposes only. All other enquiries should be sent to jbisynthesis@adelaide.edu.au.

JBI Critical Appraisal Checklist for Case Series

Reviewer: Britt Debaene, Sander Vandeweege and Hans Verstraelen Date: 16/04/2023

Author: D. Trokoudes Year: 2019 Record Number__________

	Yes	No	Unclear	Not applicable
• Were there clear criteria for inclusion in the case series?	X	☐	☐	☐
• Was the condition measured in a standard, reliable way for all participants included in the case series?	☐	☐	X	☐
• Were valid methods used for identification of the condition for all participants included in the case series?	☐	☐	X	☐
• Did the case series have consecutive inclusion of participants?	☐	☐	X	☐
• Did the case series have complete inclusion of participants?	☐	☐	X	☐
• Was there clear reporting of the demographics of the participants in the study?	X	☐	☐	☐
• Was there clear reporting of clinical information of the participants?	X	☐	☐	☐
• Were the outcomes or follow up results of cases clearly reported?	X	☐	☐	☐
• Was there clear reporting of the presenting site(s)/clinic(s) demographic information?	☐	☐	X	☐
• Was statistical analysis appropriate?	☐	☐	☐	X

Overall appraisal: Include X Exclude ☐ Seek further info ☐

Comments (Including reason for exclusion)

___

___

© JBI, 2020. All rights reserved. JBI grants use of these tools for research purposes only. All other enquiries should be sent to jbisynthesis@adelaide.edu.au.

JBI CRITICAL APPRAISAL CHECKLIST FOR CASE CONTROL STUDIES

Reviewer: Britt Debaene, Sander Vandeweege and Hans Verstraelen Date: 16/04/2023

Author: C.A. Higgins Year: 2012 Record Number__________

	Yes	No	Unclear	Not applicable
1. Were the groups comparable other than the presence of disease in cases or the absence of disease in controls?	☐	X	☐	☐
2. Were cases and controls matched appropriately?	☐	☐	☐	☐
3. Were the same criteria used for identification of cases and controls?	☐	☐	X	☐
4. Was exposure measured in a standard, valid and reliable way?	X	☐	☐	☐
5. Was exposure measured in the same way for cases and controls?	X	☐	☐	☐
6. Were confounding factors identified?	X	☐	☐	☐
7. Were strategies to deal with confounding factors stated?	X	☐	☐	☐
8. Were outcomes assessed in a standard, valid and reliable way for cases and controls?	X	☐	☐	☐
9. Was the exposure period of interest long enough to be meaningful?	☐	☐	☐	X
10. Was appropriate statistical analysis used?	X	☐	☐	☐

Overall appraisal: Include X Exclude ☐ Seek further info ☐

Comments (Including reason for exclusion)

© JBI, 2020. All rights reserved. JBI grants use of these tools for research purposes only. All other enquiries should be sent to jbisynthesis@adelaide.edu.au.
